# Excited States of Gold(I) Compounds, Luminescence and Gold-Gold Bonding

**DOI:** 10.1155/MBD.1994.459

**Published:** 1994

**Authors:** John P. Fackler, Zerihun Assefa, Jennifer M. Forward, Richard J. Staples

**Affiliations:** 1 Department of Chemistry Laboratory for Molecular Structure and Bonding Texas A&M University College Station TX 77843-3255 USA

## Abstract

It has long been established by Khan that the superoxide anion, O_2_^-^, generates singlet oxygen, O_2_^1^Δ_g_, during dismutation. Auranofin, gold-phosphine thiols, β-Carotene, and metal-sulfur compounds can rapidly quench singlet O_2_. The quenching of the O_2_^1^Δ_g_, which exists at 7752 cm^-1^ above the ground state triplet, may be due to the direct interaction of the singlet O_2_ with gold(I) or may require special ligands such as those containing sulfur coordinated to the metal. Thus we have been examining the excited state behavior of gold(I) species and the mechanisms for luminescence. Luminescence is observed under various conditions, with visible emission ranging from blue to red depending on the ligands coordinated to gold(I). Triplet state emission can be found from mononuclear three coordinate Au(I) species, including species which display this behavior in aqueous solution. A description is given of the luminescent three coordinate TPA (triazaphosphaadamantane) and TPPTS (triphenylphosphine-trisulfonate) complexes, the first examples of water soluble luminescent species of gold(I).


### EXCITED STATES OF GOLD(I) COMPOUNDS, LUMINESCENCE AND GOLD-GOLD BONDING

John P. Fackler, Jr., Zerihun Assefa, Jennifer M. Forward and Richard J. Staples

Department of Chemistry, Laboratory for Molecular Structure and Bonding, Texas A&M University, College Station, TX 77843-3255, USA

It has long been established by Khan that the superoxide anion, $O_2^-$, generates singlet oxygen, $O_2{}^1\Delta_g$, during dismutation. Auranofin, gold-phosphine thiols, β-Carotene, and metal-sulfur compounds can rapidly quench singlet $O_2$. The quenching of the $O_2{}^1\Delta_g$, which exists at 7752 $cm^{-1}$ above the ground state triplet, may be due to the direct interaction of the singlet $O_2$ with gold(I) or may require special ligands such as those containing sulfur coordinated to the metal. Thus we have been examining the excited state behavior of gold(I) species and the mechanisms for luminescence. Luminescence is observed under various conditions, with visible emission ranging from blue to red depending on the ligands coordinated to gold(I). Triplet state emission can be found from mononuclear three coordinate Au(I) species, including species which display this behavior in aqueous solution. A description is given of the luminescent three coordinate TPA (triazaphosphaadamantane) and TPPTS (triphenylphosphine-trisulfonate) complexes, the first examples of water soluble luminescent species of gold(I).

### Introduction

Khan[1] demonstrated that singlet oxygen is produced in the dismutation of superoxide, $O_2^-$, a product of dioxygen separation from transport enzymes. In healthy cells, superoxide build-up is prevented by the presence of copper and selenium containing enzymes.[2] In diseased cells, reduced levels of these enzymes have been found. Formation of the singlet state of dioxygen can be damaging to tissue through attack by this species on conjugated olefins and polynuclear aromatics and from the resulting organic peroxide formation. In "oxidative bursts" of human polymorphonuclear leukocytes both hydroxyl and oxychloro radicals can also result. These oxidative bursts are inhibited by $Au(CN)_2^-$, and by gold drugs in the presence of thiocyanate which is converted into cyanide by the leukocytes.[3] Thus it may be important to remove singlet dioxygen efficiently from cells.[4]

Emission from singlet oxygen occurs at 7752 $cm^{-1}$, leaving the molecule in the triplet ground state. Quenching of the excited singlet state may occur by energy transfer to species having compatible vibrational or electronic states, or by interactions with nearby heavy atoms which have large spin-orbit coupling.[5] For either mechanism to occur, relatively close contact must take place between the excited state oxygen and the quenching species. The solvent is the most reasonable quencher and for water there are OH vibrational overtones with appropriate energies to accommodate the quenching. In the absence of other quenchers, singlet oxygen in water returns to the ground state with a rate constant around $10^7 sec^{-1}$. In non-aqueous media[2] the rate decreases substantially with a value of $1.7x10^3$ $sec^{-1}$ having been determined in $CCl_4$. The lifetime of the singlet oxygen in cellular membranes and materials can be expected to be between these limits in the absence of other quenchers.

In view of the observation by Corey and Khan[4] that the gold drug Auranofin quenches the singlet state of oxygen in organic solvents and their suggestion that this may be an important role for gold drugs, we became interested in investigating the possibility that energy transfer might be an important mechanism for the singlet-triplet interconversion. Khan[5] has demonstrated that spin-orbit effects are real in that the lifetime of singlet oxygen in $CCl_4$ is shortened progressively in the presence of $PhYCH_3$, Y = S, Se, Te. The heavier the chalcogenide, the faster the rate of conversion. However, with the halides, PhX, X = F, Cl, Br, I, a similar quenching effect did not occur. Thus, while the heavy gold atom could function as the quencher by a spin-orbit mechanism, the heavy atom itself does not provide the quenching.

**Emission from Gold(I) Excited States**

We[6], and others[7], have observed that gold(I) complexes can display excited triplet state lifetimes in excess of $10^{-6}$ sec. These long life-times demonstrate that spin-orbit effects alone are insufficient to explain the very rapid quenching of $O_2{}^1\Delta_g$ by Auranofin. The lowest energy triplet state for Au(I) occurs about 15,000 $cm^{-1}$ above the ground state for the free ion, much too high an energy to mix effectively with the singlet delta state of dioxygen (Figure 1). Without a substantial reduction in the energy of this $^3D_3$ state, energy transfer appears unlikely as a mechanism for quenching the $O_2{}^1\Delta_g$ state. However, if the $^3D_3$ state is split by ligand field effects, LFS, as expected for a long lived (relative to nuclear motions) excited state, an energy match can occur. With ligand coordination by sulfur, one anticipates that the ligand field splitting could be 20,000 $cm^{-1}$ or more, certainly enough to lower the lowest component of the split $^3D_3$ state to the region of near 5000 $cm^{-1}$, allowing an energy match to occur between the excited state triplet of Au(I) and the singlet state of dioxygen. Thus we have been concerned with the excited state properties of Au(I) and the influence various ligands have on these electronic states.

Electronic Structure of Gold(I)

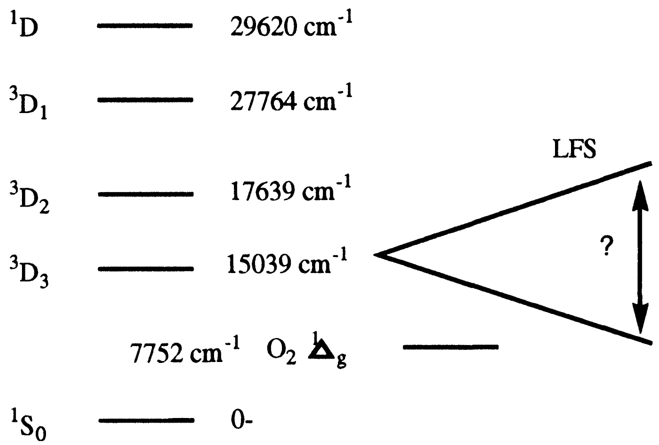

Figure 1 - Electronic structure of Au(I). The Ligand Field Splitting, (LFS), is estimated to be as much as 20,000 $cm^{-1}$

Luminescence of Au(I) compounds has been known since[8] 1970 and since 1992 for mono-nuclear Au(I) compounds in solution.[6] In mono-nuclear species which have a trigonal ligand coordination or with two coordinate complexes which have another Au(I) from another molecule in close proximity (< 3.3 Å ), metal-centered excited states are observed.[9] An example of a mononuclear trigonal complex which shows this luminescence can be seen with the tris(2-(diphenylphosphino)ethyl)amine complex, $Au(NP_3)PF_6$, Figure 2, which has a $AuP_3$ coordination and no Au-N bonding

With ligands coordinating through sulfur atoms, excitation can occur either on the ligands themselves or from ligand to metal charge transfer (LMCT) states. In the $L_2AuS$ coordinated species, the sulfur non-bonding electrons enable an n-$\pi^*$ excitation on the ligand to produce a ligand centered emission.[6c]

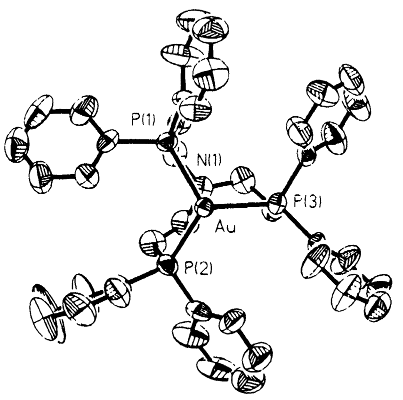

Figure 2 - The structure of the three coordinate cation tris-(2-diphenylphosphino)ethyl)amine-Au(I)

**Water Soluble Luminescent Gold(I) Complexes**

The development of phosphine ligands which can impart water solubility to metal complexes for purposes of catalysis has suggested to us that it might be possible to synthesize gold(I) complexes which are luminescent in aqueous solution. One such ligand[10] is 1,3,5-triaza-7-phosphaadamantane, TPA, Figure 3. This ligand readily forms complexes with Au(I). In addition, the nitrogen atoms on the ligand are sufficiently basic that protonation occurs below a pH of about 4.5. Thus at low pH, the ligand protonated complexes occur while at high pH, unprotonated species can be isolated. The structure of one unusual product formed from $Au(TPA)_3^-$ already has been reported.[11]

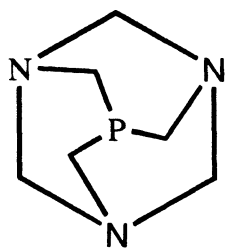

Figure 3 - Line drawing of 1,3,5-Triaza-7-PhosphaAdmantane, TPA

In addition to the (TPA)AuX, X = Cl, Br, I, Me, Ph, complexes which have been characterized structurally to date[12,13] the protonated species $(H\text{-}TPA)AuX_2$, X = Cl, Br, have been crystallographically characterized, as have the complexes $[(TPA)_2Au]Cl$, $[(H\text{-}TPA)_3(TPA)Au]Cl_4$, and $[(TPA)_4Au][PF_6].1.5HCl.2H_2O$. None of these materials are luminescent in aqueous solution. However, the $[(TPA)_3Au]Cl$ complex, which has not been crystallized to date, is strongly luminescent in the solid state and in aqueous solution above pH 4.5. Below pH 4, the complex disproportionates into the protonated tetrakis- complex and the bis- complex, neither of which is luminescent. A crystallographically characterized complex has been prepared by methylating a TPA nitrogen atom on each ligand[14]. This complex, shown in Figure 4, $[(TPA.MeI)_3Au]I$, has a trigonal planar coordination of the ligands to the metal atom with a long, 2.94 Å, Au-I distance. It is luminescent as a solid.

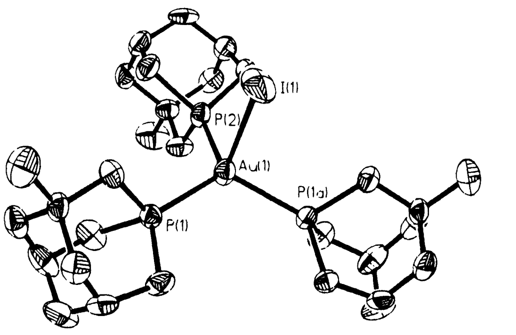

Figure 4 - Thermal ellipsoid drawing of the structure of the methylated TPA complex coordinated to Au(I), $[(TPA.MeI)_3Au]I$

By sulfonating the phenyl rings of the triphenylphosphine, it is also possible to prepare luminescent, water soluble Au(I) complexes. We have established that up to only three of these ligands can coordinate to the metal ion because of the ligands steric bulk. The visible emission spectrum of the complex is shown in Figure 5[15], a spectrum which also is sensitive to changes which occur in the solvent. Since the tetrakis- species cannot form, it is believed, and $^{31}P$ NMR results support, that the tris- complex dissociates into the bis-complex and free ligand as the polarity of the solvent diminishes. Table 1 presents some of this data including lifetimes.

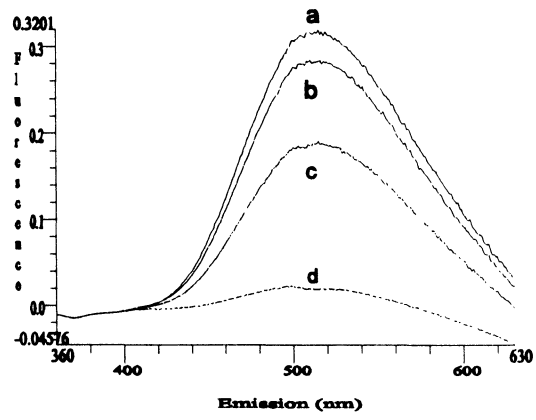

Figure 5 - Emission spectra of $(TPPTS)_3Au$, in water and water containing varying amounts of methanol. a = in water; b = +10%(v/v) MeOH; c = +20%; d = +30%. Excitation at 360 nm

| Complex | $[(TPA)_3Au]Cl$ | $(TPPTS)_3Au$ |
|---|---|---|
| $\lambda_{em}$ (nm) 78K | 517 | 492 |
| $\lambda_{em}$ (nm) 298K | 533 | 494 |
| $\lambda_{em}$ (nm) Aqueous Soln. | 547 | 513 |
| Lifetime (μsec) | 0.53 (soln.), 3.2 (solid) | 1.9,8.0 (soln.) |
| Quantum Yield | 0.069 | |

Table I. Spectroscopic data for $[(TPA)_3Au]Cl$ and $(TPPTS)_3Au$. Excitation at 360 nm.

**Multiple State Emission**

While studying the physical properties of the (TPA)AuX complexes[12] which crystallize as dimers, we observed that protonation of the TPA leads to a significant lengthening of the Au---Au distance. These complexes crystallize with a crossed "lollypop" $[(TPA)AuX]_2$ geometry. With the (TPA)AuCl complex and its protonated analogue, [(H-PTA)AuCl]Cl, it has been possible to establish that the change in the Au---Au distance is paralleled by a shift to lower energies of the visible emission. This shift is consistent with molecular orbital calculations which suggest that the HOMO-LUMO energy separation in the complex changes approximately linearly with Au-Au distance over the 3.1-3.5 angstroms. As the distance gets longer, the HOMO-LUMO gap increases. The lengthening of the Au---Au distance appears to be caused largely by the repulsive charge built up on the protonated ligands. Other distances in the complexes are nearly identical in the protonated and unprotonated species. The emission clearly is metal-based. With X = Br or I or with sulfur ligands, the electronic structure changes. LMCT is evident with these complexes. With the Br and I species, multiple state emission is observed with low energy excitation giving rise to an emission that is at a higher energy than is the metal-centered emission. With the (TPA)AuBr complex at 78 K, excitation at 320 nm produces a broad, metal-centered emission centering at 647 nm while excitation at 340 nm leads to a structured emission centered around 475 nm, Figure 6. An energy level diagram generalizing this behavior is presented in Figure 7.

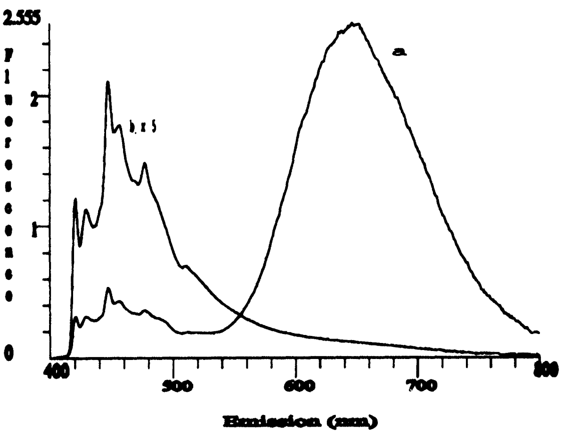

Figure 6 - Multiple state emission of (TPA)AuBr at 78K with excitation at 320 nm producing the emission at (a) 647nm and excitation at 340 nm giving the high energy emission at (b) 460 nm.

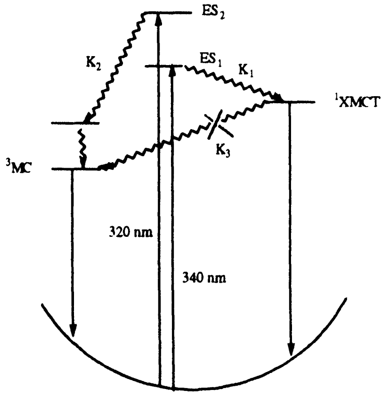

Figure 7 - An electronic state diagram which corresponds to the observed two state emission for the (TPA)AuX, X = Br,I, complexes.

**Sulphur Coordinated Complexes**

Since compounds with sulfur coordination also display the LMCT emission[16], one might ask where the metal-centered emission occurs? One possibility is that it has moved even further to low energies as suggested by the changes observed when Br is replaced by I. Alternatively, there could be a complete crossover between the LMCT and metal-centered states. With compounds which crystallize in a manner precluding any Au---Au interaction, the LMCT emission appears to occur at higher energies than with those compounds which show close, 3.1 Å, Au---Au contacts. Emission from ligand-centered states also has been observed from complexes containing triphenylphosphine ligands as shown in Figure 8. If the metal-centered, ligand field split $^3D_3$ state has been lowered so that emission is shifted to the IR (below 700 nm), it is reasonable to believe that quenching of singlet oxygen occurs by energy transfer associated with a low lying triplet state on the metal. Efforts to test this hypothesis are currently underway.

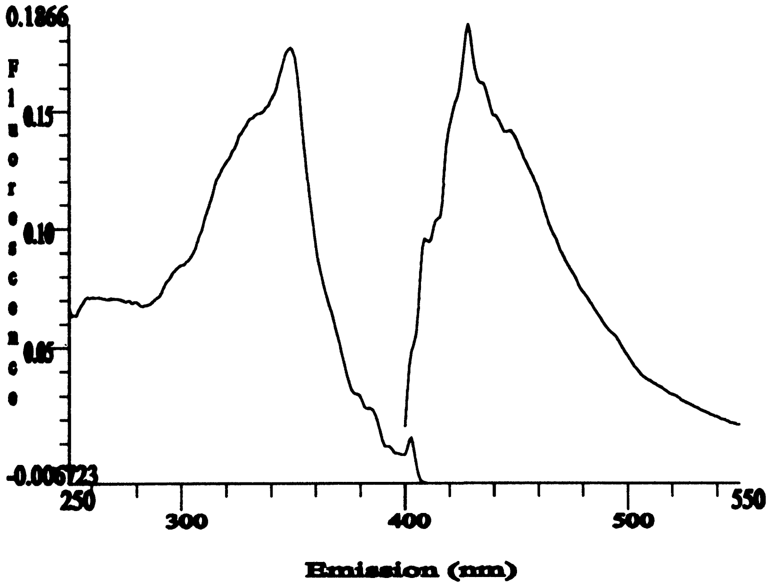

Figure 8 - The luminescence spectrum of $(Ph_3P)AuSPh(o\text{-}OMe)$, a species with a P-Au-S coordination and no Au---Au interaction in the solid state. The luminescence appears to be a fluorescence centered on the triphenylphosphine ligand.

**Acknowledgements.** The support of the U.S. National Science Foundation (9300107), the Robert A. Welch Foundation of Houston, Texas, and the Advanced Research Program of the State of Texas are gratefully acknowledged. We also thank Alice and Mitchell Bruce for preprints of their work.

**References.**

1. Khan, Ahsan U., *J. Amer. Chem. Soc.*, 1981, **103**, 6516-6517.

2. Sawyer, Donald T., *Oxygen Chemistry*, Oxford University Press, 1991.

3. Graham, Garry G.; Champion, G. David; Ziegler, John B., *Proceedings of the Third Internation[illegible] Conference on Gold and Silver in Medicine*, Shaw,III, C. Frank, ed. 1994, Milwaukee, Wisc.

4. Corey, E.J.; Mehrotra, M.M.; Khan, A.U., *Science*, 1987, **236**, 68-69.

5. Corey, E.J.; Khan, A.U.; Ha, D-C., *Tetrahedron Letters*, 1990, **31**, 1389-1392.

6. a) King, C.; Wang, J-C.; Khan, Md. N. I.; Fackler, Jr., J. P., *Inorg. Chem.,* 1989, **28**, 2145. b) King, C.; Khan, Md. N.I.; Staples. R.J.; Fackler, Jr., J.P., *Inorg. Chem.,* 1992, **31**, 3236-3238. c) Assefa, Z.; Staples, R.J.; Fackler, Jr., J.P. *Inorg. Chem.,* 1994, **33**, 2790-2798.

7. McClesky, T.M.; Gray, H.B., *Inorg. Chem.,* 1992, **31**, 1733-1734. *Chem.*1990, **29(17)**, 3203-Balch, A.L.; Fung, E.Y.; Olmstead, M.M., *Inorg.* 3207. Jaw, H.-R.C.; Savas, M.M.; Rogers, R.D.; Mason, W.R., *Inorg. Chem.,* , 1989, **28**, 1028-1037. Che, C.-M.; Kwong, H.-L.; Yam, V. W.-W.; Cho, K.-C.H., *J. Chem. Soc., Chem. Commun.*, 1989, 885-886.

8. Ziolo, R.F.; Lipton, S.; Dori, Z., *J. Chem. Soc. Chem. Commun,* 1970, 1124-1125.

9. Khan, Md. N.I.; Staples, R.J.; King, C.; Fackler, Jr., J.P.; Winpenny, R.E.P., *Inorg. Chem.,* 1993, **32**, 5800-5807.

10. Daigle, D.; Pepperman, Jr.; A.B.; Vail, S.L.; *J. Heterocycl. Chem.*, 1974, **11**, 407. DeLemo, J.R.; Trefnas, L.M.; Darensbourg, M.Y.; Majeste, R.J., *Inorg. Chem*, 1976, **15**, 815.

11. Fackler, Jr., J.P.; Staples, R.J.; Assefa, Z. *J. Chem. Soc. Chem. Commun.*, 1994, 431-432.

12. a) Assefa, Z.; McBurnett, B.G.; Staples, R.J.; Fackler, Jr., J.P.; Assman, B.; Angermaier, K., Schmidbaur, H., submitted to *Inorg. Chem.,* b) Assefa, Z.; Forward, J.M.; Staples, R.J.; Fackler, Jr., J.P., to be submitted.

13. Jones, W.B.; Yuan, J.; Narayanaswamy, R.; Young, M.A.; Elder, R.C.; Bruce A.E.; Bruce, M.R.M., submitted

14. This complex has been crystallographically characterized and will be published elsewhere.

15. Emission and excitation spectra measured on a SLM/AMINCO, model 8100 spectrofluorometer using a Xenon lamp. Spectra were corrected for instrumental response.

16. Bruce, M.R.M.; Bruce, A.E., private communication.

